# Investigating the Influence of Counterflow Regions on the Hydrodynamic Performance of Biomimetic Robotic Fish

**DOI:** 10.3390/biomimetics9080452

**Published:** 2024-07-24

**Authors:** Yanling Gong, Ming Wang, Qianchuan Zhao, Ruilong Wang, Lingchen Zuo, Xuehan Zheng, He Gao

**Affiliations:** 1 School of Information and Electrical Engineering, Shandong Jianzhu University, Jinan 250101, China; 2 Department of Automation, Tsinghua University, Beijing 100084, China; 3 Shandong Zhengchen Technology Co., Ltd., Jinan 250101, China

**Keywords:** biomimetic robotic fish, propulsive efficiency, computational fluid dynamics, countercurrent waters

## Abstract

Biomimetic robotic fish are a novel approach to studying quiet, highly agile, and efficient underwater propulsion systems, attracting significant interest from experts in robotics and engineering. These versatile robots showcase their ability to operate effectively in various water conditions. Nevertheless, the comprehension of the swimming mechanics and the evolution of the flow field of flexible robots in counterflow regions is still unknown. This paper presents a framework for the self-propulsion of robotic fish that imitates biological characteristics. The method utilizes computational fluid dynamics to analyze the hydrodynamic efficiency of the organisms at different frequencies of tail movement, under both still and opposing flow circumstances. Moreover, this study clarifies the mechanisms that explain how changes in the aquatic environment affect the speed and efficiency of propulsion. It also examines the most effective swimming tactics for places with counterflow. The results suggest that the propulsion effectiveness of robotic fish in counterflow locations does not consistently correspond to various tail-beat frequencies. By utilizing vorticity maps, a comparative analysis can identify situations when counterflow zones improve the efficiency of propulsion.

## 1. Introduction

Over the past few years, there has been a growing global interest in the exploration and scientific study of marine resources [1]. As a result, there has been a rapid advancement in the theory and technology of underwater robots. The emergence of biomimetic robotic fish as a novel sort of underwater vehicle has garnered significant interest among engineers and scientists. With the rapid development of manufacturing, control, and sensing technologies, biomimetic robotic fish have become more and more important in the fields of ocean exploration, underwater rescue, and water quality monitoring [2,3,4,5]. Bionic robotic fish can not only explore the ocean and discover new species but can also be used for the salvage and construction of undersea facilities. Compared to other devices, bionic robotic fish exhibit higher levels of performance when working in hazardous environments [6]. For example. soft bionic robotic fish are better adapted to the complex environments of coral areas [7]. Fish robots have recently become increasingly popular for a variety of applications, including maritime research, military operations, and environmental protection. These applications require high-performance autonomous underwater vehicles that offer significant advantages in propulsive efficiency and flexible maneuverability in particular [8,9].

The biomimetic robotic fish’s design concept allows for efficient, silent, and extremely nimble swimming underwater. This is accomplished by imitating the locomotion abilities of fish in their natural environment [10,11]. Biomimetic robotic fish provide several benefits, including increased efficiency, improved agility, decreased noise, and the integration of propulsion and control systems. Furthermore, fish have the capability to utilize their tail fins to exploit the energy from nearby turbulence, hence augmenting their swimming proficiency [12,13]. This swimming pattern is called the Labriform model [14]. Fish can enhance their swimming efficiency by many adaptations, including reducing drag by having smooth surface structures [15], utilizing the energy from currents produced by nearby fish [16], modifying the flexibility of their tails [17], and optimizing their swimming patterns [18,19].

Fish are renowned for producing minimal wakes and adapting adeptly to complex aquatic environments. Consequently, extensive bionic studies have been dedicated to understanding fish locomotion [20]. Research has revealed that fish generate thrust by orchestrating the movements of their fins to create a well-structured flow field. This intricate process is accompanied by the generation of unsteady vortices [21]. During fin oscillations, these vortices can adhere to the fin surface, thereby delaying stall and enhancing lift [22]. These findings underscore the pivotal role of vortices generated during fish movements in facilitating thrust production and improving overall efficiency. Robotic fish offer advantages in motion control and structural parameters compared to biological experiments [23]. Consequently, they serve as simplified models for researching the swimming principles of fish. Moreover, by replicating fish morphology and propulsion mechanisms, robotic fish can achieve efficient swimming and enhance their performance in complex underwater environments.

To enhance the stability of movement and precision of control in robotic fish swimming, it is essential to analyze the hydrodynamic characteristics of their swimming motion and develop a dynamic model for motion control. Currently, numerous studies focus on dynamic modeling, which forms the foundation for controlling and optimizing robotic fish [24,25]. From a bionics perspective, it is imperative to account for the effect of vortices on motion to establish an accurate dynamic model for robotic fish. In the field of robotic fish, many researchers have used CFD to explore the effects of fin flexibility or stiffness [26,27], to evaluate hydrodynamic coefficients [28], and to optimize robot gait [29,30]. Other applications include validating analytical models [31], obtaining fluctuation-induced fluid effects [32], optimizing fish morphology [33], and research on the control of swimming mechanisms in forming fish shoals [34]. Ming et al. [35] coupled a 3D CFD model to the motion of a fish and computed the distribution of torque and power. Zhao and Dou [36] performed a combined fluctuation–motion model of a bionic fish (CUMP) that was numerically simulated. They also investigated the effect of the spatial distance between the fluctuating and fluttering wings on the propulsive performance. Li et al. [37] combined CFD and multibody dynamics (MBD) to investigate the self-propelled motion of the fish during the acceleration and quasi-steady state phases. Gao [38] numerically simulated the hydrodynamic performance of a traveling wave by using the mesh deformation technique. Sun [39] established a simplified two-dimensional model based on CFD to investigate the effect of the leading edge shape on the hydrodynamic properties of the wave type. Although the above scholars have conducted a lot of analysis on the swimming of bionic fish, based on CFD, the comparison of the propulsion efficiency of self-propelled bionic robotic fish in countercurrent waters as well as the analysis of eddy currents still needs to be continuously and deeply investigated.

To establish the propulsive efficiency of a bionic robotic fish, Triantafyllou et al. [40,41] proposed several calculation methods, primarily based on the Froude efficiency or quasi-propulsion efficiency and utilizing the transportation cost (Cost of Transport, COT). The Froude efficiency quantifies the ratio of useful power to total power. Specifically, for steady-swimming fish, the Froude efficiency delineates the useful work, such as thrust, from reactive work, like lateral tail swing, to determine the proportion of energy effectively converted to forward swimming. Borazjani et al. [42] conducted an in-depth investigation into the definition and calculation of hydrodynamics, power, and efficiency, particularly emphasizing the significance of the Froude efficiency in rate determination. Tian et al. [43] conducted numerical analyses of a pair of female fish swimming in a uniform flow and observed enhanced swimming efficiency when the fish were arranged in series or staggered formation. Their examination of the wake vortex structure revealed the crucial role of eddy current control in tail fish performance improvement. Zou et al. [44] propose a discrete vortex method; this approach offers an effective means to incorporate vortex dynamics into dynamic modeling. They suggest potential benefits from enhancing the anti-Kármán vortex street or reducing the Kármán vortex street. These studies collectively emphasize the importance of understanding and quantifying propulsion efficiency in robotic fish, shedding light on various methodologies and mechanisms for improving their performance in different flow environments. However, there remains a need for further investigation into the comparison of propulsion efficiency in self-propelled bionic robotic fish under countercurrent waters as well as a deeper analysis of eddy currents.

Water flow is a key factor affecting the hydrodynamics of bionic robotic fish. It is well known that fish can derive energy from vortices when passing through turbulence [45], but head-on flow has a negative impact on propulsive performance [46]. Flow perturbations can alter the course of vortex shedding, which in turn affects hydrodynamics [47]. The goal of this study is to employ numerical simulation methods to examine the hydrodynamic coefficients of a robotic fish inspired by nature, both in still water and in water with opposing currents.

The main contributions of this paper are as follows:

(1) A bionic robotic fish model, capable of self-propelled motion, has been created. The fish is powered by a user-defined function (UDF).
(2) This study presents a method to calculate propulsive efficiency in order to analyze and compare the propulsive efficiency and hydrodynamic parameters of a bionic robotic fish. This study investigates the effects of modifying the tail-beat frequency of the fish under hydrostatic and countercurrent conditions.
(3) A more intuitive post-processing method confirms the propulsive efficiency through quantitative analysis of the anti-Kármán vortex. It is concluded that a specific countercurrent water environment aids in the forward self-propulsive swimming of the bionic robotic fish. Additionally, the bionic robotic fish can enhance its propulsive speed by harnessing the power of the water.

The remainder of the paper is organized as follows. Section 2 describes the classical fish body wave model with the establishment of an improved self-propulsion model for the bionic robotic fish. Section 3 provides the methodology and boundary conditions for numerical simulation. Section 4 presents the analysis and discussion of the simulation results. Finally, a summary and ongoing work has been provided in Section 5.

## 2. Model Building and Calculation Method

### 2.1. Kinematic Model

**Figure 1:**
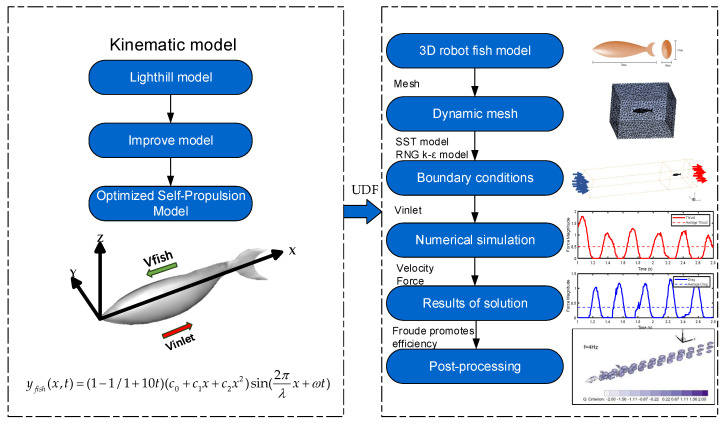
Flow chart of hydrodynamic numerical simulation.

A pattern of body fluctuations is presented by a fish from head to tail as it swims. The kinematic model of this paper is shown in Figure 1. Lighthill’s proposed body wave model for trevally fish is as follows [48]:

(1) $$\left\{\begin{array}{l} y_{body}(x,t)=(c_{1}x+c_{2}x^{2})\sin(\frac{2\pi }{\lambda }x+\omega t)\\ \omega =2\pi f \end{array}\right.$$

where $y_{body}(x,t)$ is the amplitude of the fish’s oscillation along the vertical centerline, x is the displacement of the fish along the direction from the tail to the head, $c_{1}$ and $c_{2}$ are the primary and secondary coefficients of the fish envelope, and λ is the wavelength of the fish. The model describes the ideal swimming pattern of a bionic fish, assuming that the head is rigid and does not oscillate to either side of the axis. Although the description of this model is very close to the real fish’s swimming state, its performance is still very different from that of a fish. In actual fish swimming, head bobbing caused by inertia and reaction forces can be biologically suppressed.

The bionic robot fish also has an inertia force and a reaction force when swimming. In addition, head bobbing cannot be completely eliminated due to factors such as fish size, scale configuration, and processing errors. Therefore, using the above kinematic model to describe the motion of the bionic fish is not completely accurate. The researchers modified the model by converting the swimming of the fish into a function of the tail motion relative to the head [49]. The improved model is represented as follows:

(2) $$y_{fish}(x,t)=(c_{1}x+c_{2}x^{2})\sin(\frac{2\pi }{\lambda }x+\omega t)-c_{1}x\sin(\omega t)$$

where $c_{1}\sin(\omega t)$ is the value of the first order derivative of $y_{fish}(x,t)$ at x=0. The improved model has a more optimized swimming attitude, but it is required that the bionic robotic fish can perform self-propelled swimming that lacks a stationary, accelerated to stable propulsion process.

Finally, considering the small head bobbing of the bionic fish, the initial model is optimized, and a constant term is added to the amplitude envelope [50]. To realize that self-propelled swimming is possible at time t=0, a time coefficient is added in front of the traveling wave function. The time coefficient is $1-1/\left(1+10t\right)$. At t=0, this term becomes 0, ensuring that the bionic robot fish does not swim at the initial moment. As time passes and t increases, the term $\left(1+10t\right)$ grows larger, causing $1/\left(1+10t\right)$ to decrease toward zero. Consequently, the time coefficient $1-1/\left(1+10t\right)$ approaches 1. This means the time coefficient mainly affects the initial moment of swimming, allowing the fish to start from rest and gradually reach its full swimming motion. The optimized self-propulsion model is expressed as follows:

(3) $$y_{fish}(x,t)=\left(1-\frac{1}{1+10t}\right)\left(c_{0}+c_{1}x+c_{2}x^{2}\right)\sin\left(\frac{2\pi }{\lambda }x+\omega t\right)$$

where $c_{0}$ is the constant term of the envelope. To facilitate the comparison of research results with the reference literature, we define the parameters as follows in the self-propulsion model: $c_{0}$ = 0.02, $c_{1}$ = −0.08, $c_{2}$ = 0.16, λ = 0.95, f = 1.0 Hz [51].

**Figure 2:**
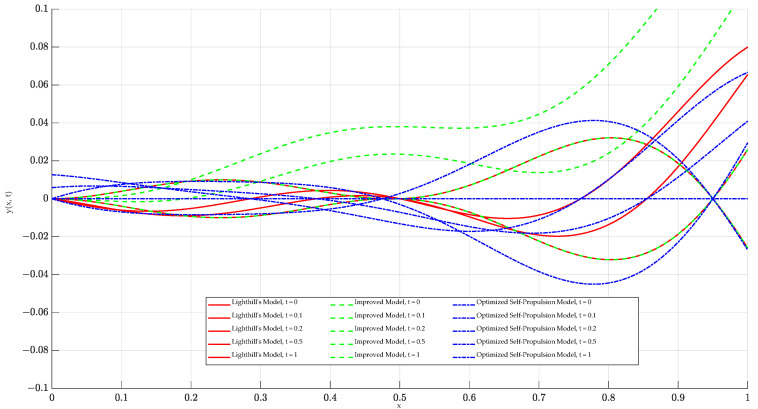
Comparison of different models for bionic fish at different moments.

Based on MATLAB simulations of the initial model, the improved model, and the self-propulsion model, the analysis results are shown in Figure 2. Figure 2 presents the fish body wave curves at different time points for each of the three models. In the initial model (Lighthill model), the head and middle of the body show no oscillation, while the back and tail exhibit significant oscillation. To more closely mimic the swimming of real fish, the improved model features slight oscillations in the front and tail of the body, with more pronounced oscillations in the back, but the head still remains stationary. In the final self-propulsion model, both the head and the front part of the body show slight oscillations, with the back and tail following suit with more substantial oscillations. The detailed differences among the three models at different time points are illustrated in the figure. The wave pattern of the self-propulsion model is similar to the initial model but shows significant differences from the improved model, resulting in a smoother body motion. In summary, the motion posture simulation indicates that the self-propulsion model better aligns with the swimming mode of bionic fish.

### 2.2. Numerical Simulation Model

In this paper, a 3D simulation area is constructed to simulate the swimming process of the bionic robot fish, which swings along the Y-axis and swims along the X-axis in the negative direction, as shown in the right panel of Figure 1. Two-dimensional modeling and numerical simulations were performed on a Lenovo desktop computer. The computer was configured with a 16-core Intel(R) Core(TM) i7-113700 CPU@5.20 GHz and 32.0 GB RAM, sourced from Dell (China) Ltd., Fujian, China.

For the boundary conditions, the inlet type is set as velocity inlet, the outlet type is set as outflow, and the wall type inside the swimming region is set as a no-slip wall. The total length of the bionic robot fish is *BL*, including the head length. For the scale of the computational region, the total length, total width, and total height were taken as *L* = 15 *BL*, *W* = 2 *BL*, and *H* = 2 *BL*, respectively. For the mesh, the finest mesh spacing was *BL*/116 in the region near the fish-like model, and the coarsest mesh spacing was *BL*/8 in the other regions.

For the calculations, the hydrodynamic simulations were performed in ANSYS-Fluent 2020 R2 (ANSYS Inc., Canonsburg, PA, USA) software to create a global numerical model in the Cartesian coordinate system. The Navier–Stokes equations are represented in the Cartesian coordinate system as follows [52]:

(4) $$\left\{\begin{array}{l} \frac{\partial (\rho u_{x})}{\partial t}+\nabla \cdot (\rho u_{x}\vec{u})=F_{x}-\frac{\partial p}{\partial x}+\frac{\partial \tau_{xx}}{\partial x}+\frac{\partial \tau_{yx}}{\partial y}+\frac{\partial \tau_{zx}}{\partial z}\\ \frac{\partial (\rho u_{y})}{\partial t}+\nabla \cdot (\rho u_{y}\vec{u})=F_{y}-\frac{\partial p}{\partial y}+\frac{\partial \tau_{xy}}{\partial x}+\frac{\partial \tau_{yy}}{\partial y}+\frac{\partial \tau_{zy}}{\partial z}\\ \frac{\partial (\rho u_{z})}{\partial t}+\nabla \cdot (\rho u_{z}\vec{u})=F_{z}-\frac{\partial p}{\partial x}+\frac{\partial \tau_{xz}}{\partial x}+\frac{\partial \tau_{yz}}{\partial y}+\frac{\partial \tau_{zz}}{\partial z} \end{array}\right.$$

where $u_{x}$, $u_{y}$, $u_{z}$, respectively, and x, y, z are the velocity components of the direction at time t, ρ is the fluid density, τ is the stress tensor, and $F_{x}$, $F_{y}$, and $F_{z}$ are the forces applied to the object.

Turbulence modeling is based on the Reynolds mean NS equation, and there are three general turbulence models: the standard model, SST, and BSL. All three models are similar in form to transport equations ε. After comprehensive comparison, the RNG k−ε model better takes into account the effect of the turbulent structure by using eddy viscosity correction. In the simulation of bionic robotic fish, the eddy structure has an important effect on, among other things, the propulsion efficiency, considering that this correction provides a more comprehensive characterization of the turbulence. Moreover, SST is less computationally intensive and more suitable for multiple computational simulations to produce multiple sets of valid values. The RNG k−ε model equations and standard formulas are given below:

(5) $$\frac{\partial (\rho k)}{\partial t}+\frac{\partial (\rho ku_{i})}{\partial x_{i}}=\frac{\partial }{\partial x_{j}}(\alpha_{\varepsilon }\mu_{eff}\frac{\partial k}{\partial x_{j}})+G_{k}+\rho \varepsilon$$

(6) $$\frac{\partial (\rho k)}{\partial t}+\frac{\partial (\rho ku_{i})}{\partial x_{i}}=\frac{\partial }{\partial x_{j}}(\alpha_{\varepsilon }\mu_{eff}\frac{\partial k}{\partial x_{j}})+\frac{\varepsilon }{k}C_{1\varepsilon }^{*}G_{k}-C_{2\varepsilon \rho }\frac{\varepsilon^{2}}{k}$$

(7) $$\mu_{t}=\rho C_{\mu }\frac{k^{2}}{\varepsilon },\mu_{eff}=\mu +\mu_{t}$$

where $\mu_{t}$ is the turbulent viscosity, ε is the turbulent dissipation rate, k is the turbulent kinetic energy, ρ is the fluid density, $G_{k}$ is the generation term of turbulent kinetic energy k due to the mean velocity gradient, $G_{b}$ is the generation term of turbulent kinetic energy due to buoyancy, and $C_{1\varepsilon }$ and $C_{2\varepsilon }$ are the empirical coefficients. The empirical constants are $C_{\mu }$ = 0.0845, $\partial_{k}$ = $\alpha_{\varepsilon }$ = 1.39, $C_{1\varepsilon }$ = 1.42, and $C_{2\varepsilon }$ = 1.68 for each calculation.

Finally, the pressure–velocity coupling analysis was carried out by the SIMPLE semi-implicit method using the pressure-correction equation and the velocity-correction equation. In addition, the turbulent kinetic energy and turbulent dissipation rate are both in first-order windward format to speed up the calculation.

### 2.3. Calculation of Propulsion Force and Swimming Propulsion Efficiency

The definition of the efficiency of fish swimming is controversial and ambiguous. The work of Borazjani and Sotiropoulos [53] proposed a method of varying the Strahl number St while maintaining a constant swimming velocity *U* and Reynolds number *Re*, simulating the flow induced by a model fish. The model fish is attached to and dragged by a rigid tether that translates the fish through a stationary fluid at a given constant velocity *U*. The force acting on the fish body by the current is set to be *F*. If *F* = 0, the hypothetical tether absorbs the excess force. Thus, the net force exerted on the fish is zero, satisfying the assumption of constant swimming speed. In these cases, if the hypothetical tether is momentarily cut, the fish will accelerate or decelerate under the influence of the additional force *F*. In this case, if the hypothetical tether is cut, the fish will also swim at a constant speed *U*. The fish can follow this process to maintain formation and simulate natural “free swimming” conditions.

Based on the above thesis, it follows that the Froude efficiency holds when the fish swims into a “free swimming” state, so when the thrust and hydrodynamic drag are exactly equal, the Froude efficiency, defined based on the net force in the mean swimming direction, is zero. Further, it is useful to define the Froude propulsive efficiency for uniform inline swimming based on thrust as follows:

(8) $$\eta =\frac{\overset{-}{T}U}{\overset{-}{T}U+\overset{-}{P_{Y}}}$$

where $\overset{-}{T}$ is the mean value of swimming cycle thrust, U is the steady state swimming speed, and $\overset{-}{P_{Y}}$ is the mean value of swimming cycle power loss due to lateral fluctuations.

The Froude propulsive efficiency given by Equation (8) can also be calculated directly from the CFD simulation results; however, to do this, we first need to define and develop a method for calculating thrust and drag. This is because it is not simple to define a fish-like swim, since in this case, the propulsion is the body of the fish itself, which generates thrust along with drag.

In numerical simulations, the bionic robotic fish moves from static to steady motion along the swimming direction until the thrust force is approximately equal to the drag force, which is a continuous process. The fluid force *F* along the x-negative direction can be calculated by integrating the pressure and viscous forces on the fish as follows.

In the simulations presented here, the instantaneous hydrodynamic component along the negative direction of the axis, denoted *F*, can be easily calculated by integrating the pressure and viscous forces acting on the object (where repeating the index implies summation):

(9) $$F(t)=\int_{s}\left(pn_{x}-\tau_{xj}n_{j}\right)dS$$

where p and τ are the pressure tensor and viscous stress tensor, $n_{j}$ is the jth component of the unit normal vector on dS, $n_{x}$ is the unit vector in the x direction, and S is the body surface area of the robotic fish.

(10) $$C_{F}=\frac{F(t)}{0.5\rho U_{s}^{2}L^{2}}$$

*F*(*t*) is the instantaneous net hydrodynamic force provided in Equation (9), ρ is the fluid density, $U_{S}$ is the swimming velocity, and L is the fish length. In order to separate the contributions of thrust *T*(*t*) and drag *D*(*t*), the net instantaneous force *F*(*t*) can be decomposed as follows:

(11) $$T(t)=0.5F(t)+0.5\int_{s}\left(|pn_{x}|-|\tau_{xj}n_{j}|\right)dS$$

(12) $$D(t)=-0.5F(t)+0.5\int_{s}\left(|pn_{x}|-|\tau_{xj}n_{j}|\right)dS$$

Decomposing the net force according to Equations (11) and (12) above, when $C_{F}$ > 0, T(t)>D(t), when the thrust force exceeds the drag force and the net force acting on the body is in the same direction as the fish’s motion. For ease of discussion, we will refer to this case as a thrust-type net force. Similarly, in the case of $C_{F}<0$, T(t)<D(t) will be referred to as the net force being of the resistance type.

In addition, the dimensionless thrust $C_{T}$ and drag coefficients $C_{D}$ in the swimming direction can be calculated as follows:

(13) $$C_{T}=\frac{T(t)}{0.5\rho U_{s}^{2}L^{2}}$$

(14) $$C_{D}=\frac{D(t)}{0.5\rho U_{s}^{2}L^{2}}$$

The swimming power loss due to lateral body fluctuations can be calculated as follows:

(15) $$P_{y}(t)=\int_{s}(pn_{y}-\tau_{yj}n_{j})u_{y}dS$$

where $U_{y}$ is the transverse component of the object’s velocity of motion.

The dimensionless transverse power loss coefficient can be defined as:

(16) $$C_{p}=\frac{P_{y}}{0.5\rho U_{s}^{3}L^{2}}$$

The effects of changes in the disturbed water flow in the swimming waters of the bionic robotic fish on the Froude efficiency and hydrodynamic coefficients are discussed in the following part of this study.

### 2.4. Preparation and Validation of Numerical Simulations

To evaluate the impact of the mesh size on numerical simulation results, the study divided the mesh into three levels, keeping the background mesh size constant while altering the foreground and fish body mesh sizes. Despite consistent simulation parameters across all three meshes, the results revealed minimal differences among them. However, employing a coarse mesh introduced dynamic mesh distortions during calculations, leading to instability in the solution process.

Moreover, refining the mesh beyond a certain point consumed excessive computing resources without significantly enhancing simulation outcomes. Consequently, the paper opted for a more reasonable medium mesh size to balance computational efficiency and result accuracy. Maintaining mesh consistency while numerically simulating self-propelled motion with varying parameters eliminated the influence of mesh differences on simulation outcomes. Table 1 summarizes the three mesh sizes and their corresponding simulation results, with the assumption that the fine-mesh calculation results are accurate, yielding an error of 0.

## 3. Simulation Results Analysis

### 3.1. Effects of Stillwater and Countercurrent Water Environments on Swimmers’ Motor Performance

Four simulation experiments were conducted to investigate the effects of different tail-beat frequency variations on the motion state of the bionic robotic fish. Four tail-beat frequencies were selected in the experiments, *f* = 1.0 Hz, 2.0 Hz, 3.0 Hz, and 4.0 Hz, and two water environments were considered: still water and countercurrent. The flow velocity of the countercurrent waters is *V_inlet_* = 0.05 m/s. In the experiments, when the swimming speed of the bionic robotic fish reaches a critical value, i.e., the speed fluctuates slightly up and down but the overall trend remains smooth, we define it as a quasi-steady state. Through these simulation experiments, we can gain a deeper understanding of the effects of different tail-beat frequencies on the motion state of the bionic robotic fish.

#### 3.1.1. Forward Speed

**Figure 3:**
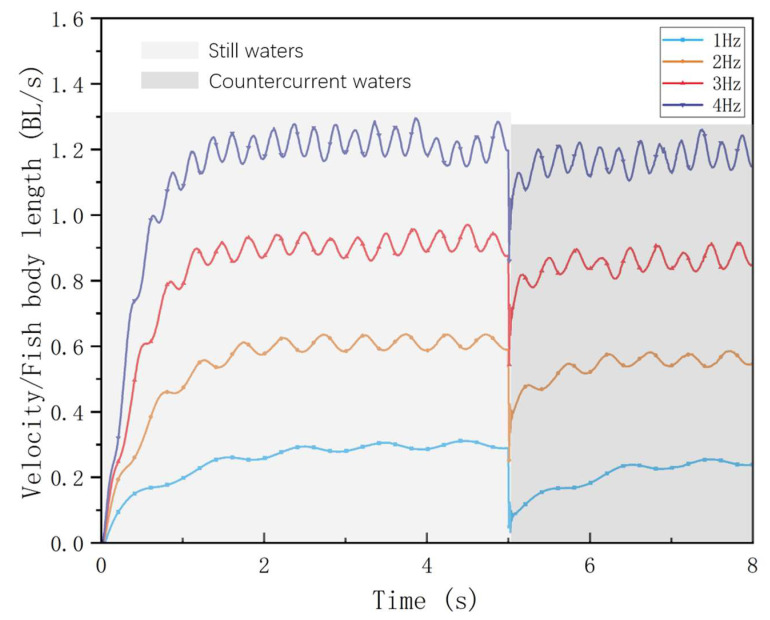
Robotic fish in still water and countercurrent waters at different tail-beat frequencies.

**Figure 4:**
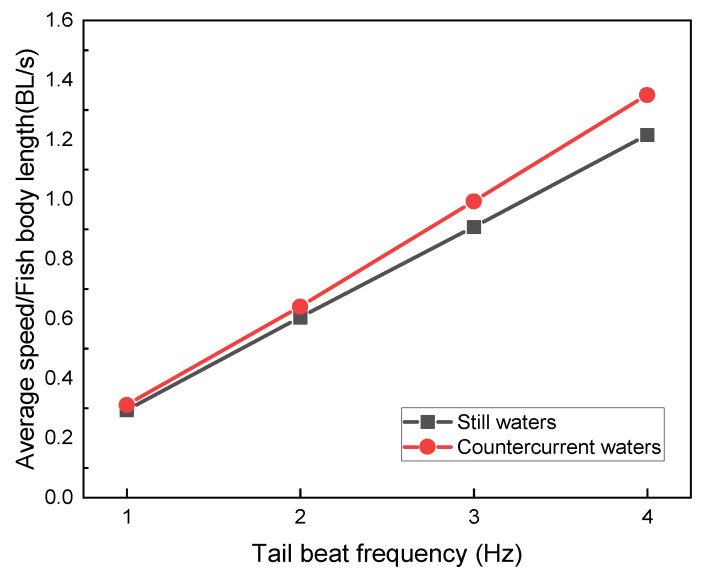
Comparison of mean speeds of robotic fish in still water and countercurrent waters at different tail-beat frequencies.

The variation in the self-propelled swimming speed of the bionic robotic fish is shown in Figure 3 and Figure 4, which is affected by the tail-beat frequency and inlet flow rate. The swimming direction speed is expressed in units of BL/s (body length per second).

From the simulation results, the swimming speeds in the countercurrent perturbed current environment all decreased relative to the static water environment. By calculating the acceleration phase time of the swimming process at each frequency, as shown in Figure 4, we found that the higher the frequency, the shorter the acceleration phase time, when the bionic robotic fish was initiated from a velocity of 0 BL/s. Subsequently, after adding the countercurrent perturbation, it can be observed that the shortest acceleration phase times against the same perturbation are at *f* = 2.0 Hz and 3.0 Hz. In contrast, the acceleration phase time at the high-frequency *f* = 4.0 Hz is not much different from that at the low-frequency *f* = 1.0 Hz.

#### 3.1.2. Froude Efficiency and Hydrodynamic Coefficients

Utilizing Froude efficiency analyses, the peak efficiencies were determined to occur at wake-beat frequencies of *f* = 2.0 Hz and *f* = 3.0 Hz. Notably, at *f* = 3.0 Hz, the propulsive efficiency was observed to be marginally augmented even in the presence of countercurrent flow. This augmentation indicates that a wake-beat frequency of *f* = 3.0 Hz optimizes vortex dynamics in countercurrent conditions.

**Figure 5:**
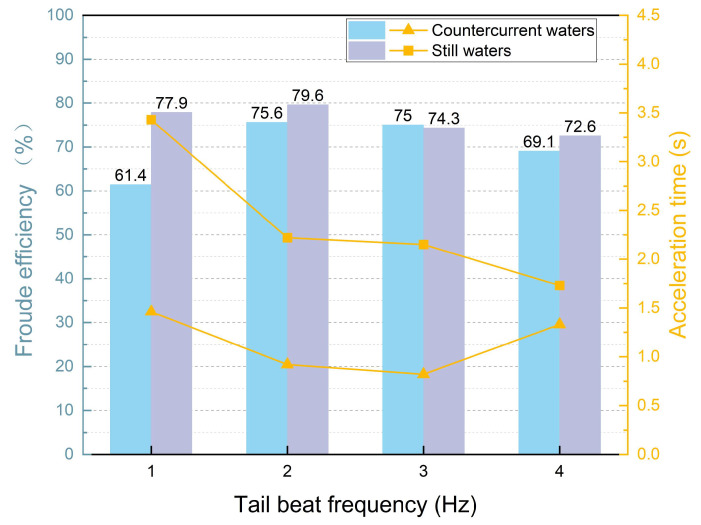
The variation in acceleration time and Froude’s efficiency of bionic robotic fish in still water and countercurrent waters with different tail-beat frequencies.

By comprehensively analyzing the acceleration time and Froude efficiency as shown in Figure 5, it can be found that, in still water, the acceleration time of the bionic robotic fish decreases as the tail-beat frequency increases, which indicates that the higher the tail-beat frequency, the faster the acceleration. However, when entering countercurrent conditions, the bionic robotic fish with a tail-beat frequency of 4 Hz exhibits the longest acceleration time, which is consistent with a decrease in its Froude efficiency. This finding suggests that higher tail-beat frequencies do not necessarily translate into higher propulsive efficiencies under certain circumstances.

**Figure 6:**
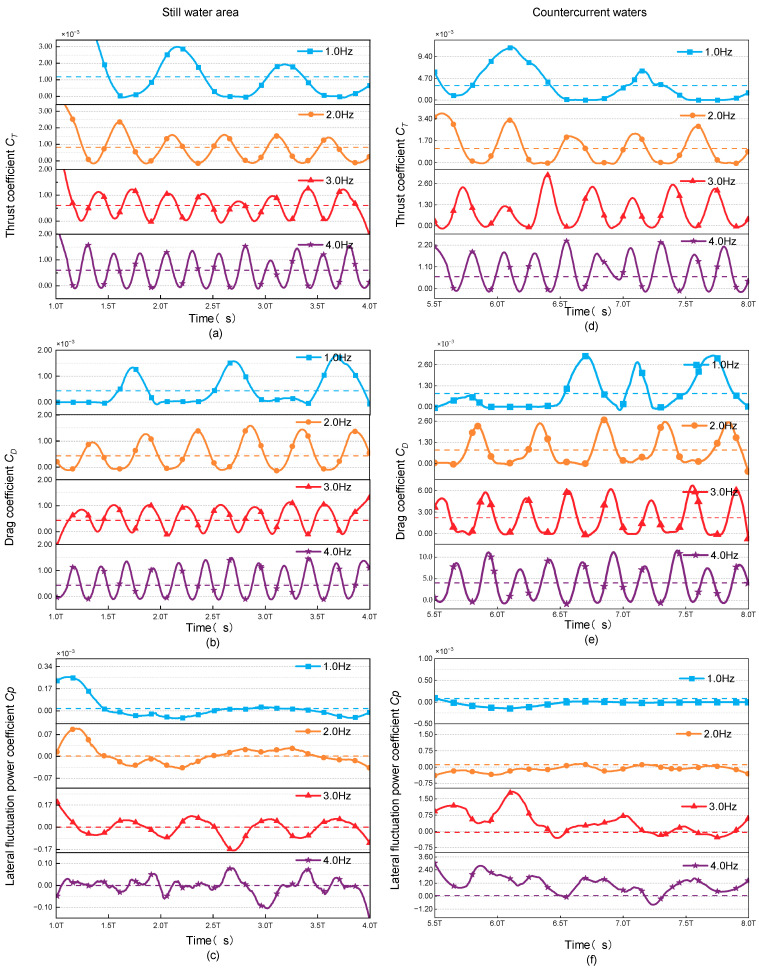
Hydrodynamic coefficients of the swimmer at different tail-beat frequencies in still water as the (**a**) Thrust coefficient *C_T_*; (**b**) Drag coefficient *C_D_*; (**c**) Lateral fluctuation power coefficient *C_p_* and in countercurrent waters as the (**d**) Thrust coefficient *C_T_*; (**e**) Drag coefficient *C_D_*; (**f**) Lateral fluctuation power coefficient *C_p_*.

A comparative evaluation of the variations in hydrodynamic coefficients between the datasets depicted in Figure 6a–c and Figure 6d–f revealed that the transverse fluctuation frequency exhibits the greatest influence on these coefficients. In countercurrent conditions, the transverse fluctuation frequency tends to diminish, approaching a near-zero value. This reduction in lateral fluctuations contributes significantly to an enhancement in the Froude efficiency.

### 3.2. Inverse Kármán Vortex Flow-Field Analysis

In the wake vortex region of a moving object, there is an aggregation of vorticity flowing down from the boundary layer of the object surface. When flow separation occurs at the surface of the object, there is also a separation of vortices into the wake region. In this region, the viscous effect is so significant that the wake vortex will continue to dissipate its mechanical energy, creating a low-pressure region, which results in differential pressure drag. However, the fish’s tail vortex control is skillfully performed by the oscillation of the caudal fin during swimming, effectively preventing the flow separation from occurring and making the trajectory region narrower. With each caudal fin oscillation, two vortices are desiccated from the trailing edge, creating a vortex street phenomenon, in which the direction of a particular vortex is reversed [54]. It provides a new perspective on our understanding of the mechanisms of fish swimming and demonstrates how organisms in nature use physical principles to optimize their movements.

The steering of this vortex is opposite to that of the familiar Kármán vortex; hence, the name reverses the Kármán vortex. In this type of vortex street, the top side is a counterclockwise vortex, and the bottom side is a clockwise vortex. A backward jet is induced between these two vortices, adding thrust to the fish’s body and helping it swim more efficiently.

**Figure 7:**
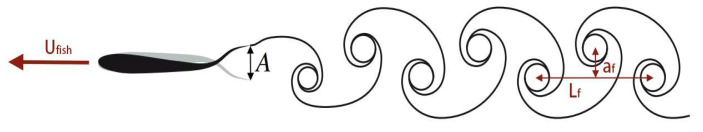
Schematic diagram of a fish swimming, which reverses the Kármán vortex street.

In the study of the vortex street structure of the tail track of the bionic robotic fish, the vortex nuclei were extracted by Tecplot software 360 EX 2021 R1, and the longitudinal spacing between the two vortex nuclei, *L_f_*, as well as the transverse distance, *a_f_*, between the vortex nuclei on both sides were measured, and *A* in Figure 7 represents the peak amplitude of the caudal fin, and *U_fish_* is the average swimming speed of the fish with arrows denoting the direction in which the fish swam.

**Figure 8:**
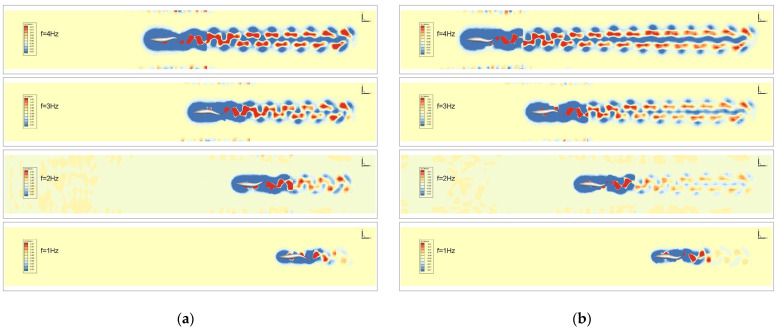
Comparison of flow-field isovorticity maps under different tail-beat frequencies: (**a**) still water; (**b**) countercurrent waters.

By comparing the isovorticograms of the flow field of the bionic robotic fish at different tail-beat frequencies, it is found that the vortex street spreads more rapidly with the increase in the tail-beat frequency, and the vortex street, which is composed of compact leading and trailing vortexes at 1 Hz, is dispersed into two vortex streets at 4 Hz. Subsequently, the distance between the vortex streets is wider in the clear water compared with the countercurrent water, which means that the two rows of vortices induce successive jets, and the propulsive force on the bionic fish increases, as shown in Figure 8.

Based on the quantitative comparison detailed in Table 2 and Table 3, it is evident that the tail-beat frequency of the bionic robotic fish has a direct impact on its swimming performance. As the tail-beat frequency increases, the longitudinal distance *L_f_* between the vortex nuclei in the wake vortex street also grows, indicating a larger forward propulsion force and, consequently, a faster swimming speed for the robotic fish.

Notably, at higher tail-beat frequencies, the transverse distance between the vortex nuclei remains relatively stable, signifying a consistent vortex formation pattern. However, at lower frequencies, *a_f_* decreases, aligning with previous findings that revealed a higher Froude propulsion efficiency at 1 Hz compared to 2 Hz in still-water conditions.

In countercurrent environments, the data reveal a particularly interesting trend. At a tail-beat frequency of 3 Hz, *L_f_* reaches its maximum value, while a_f_ is minimized. This combination results in an optimal vortex street configuration, leading to a relatively superior propulsive force and, thus, the highest Froude propulsion efficiency observed at 3 Hz in countercurrent conditions.

**Figure 9:**
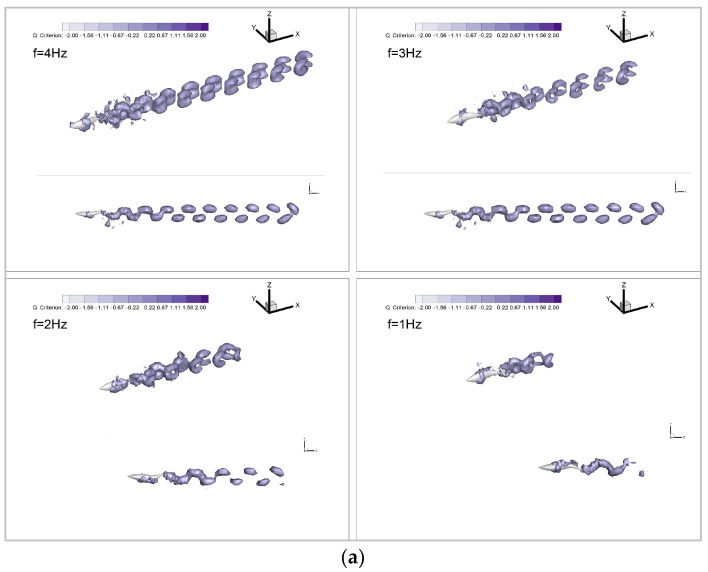

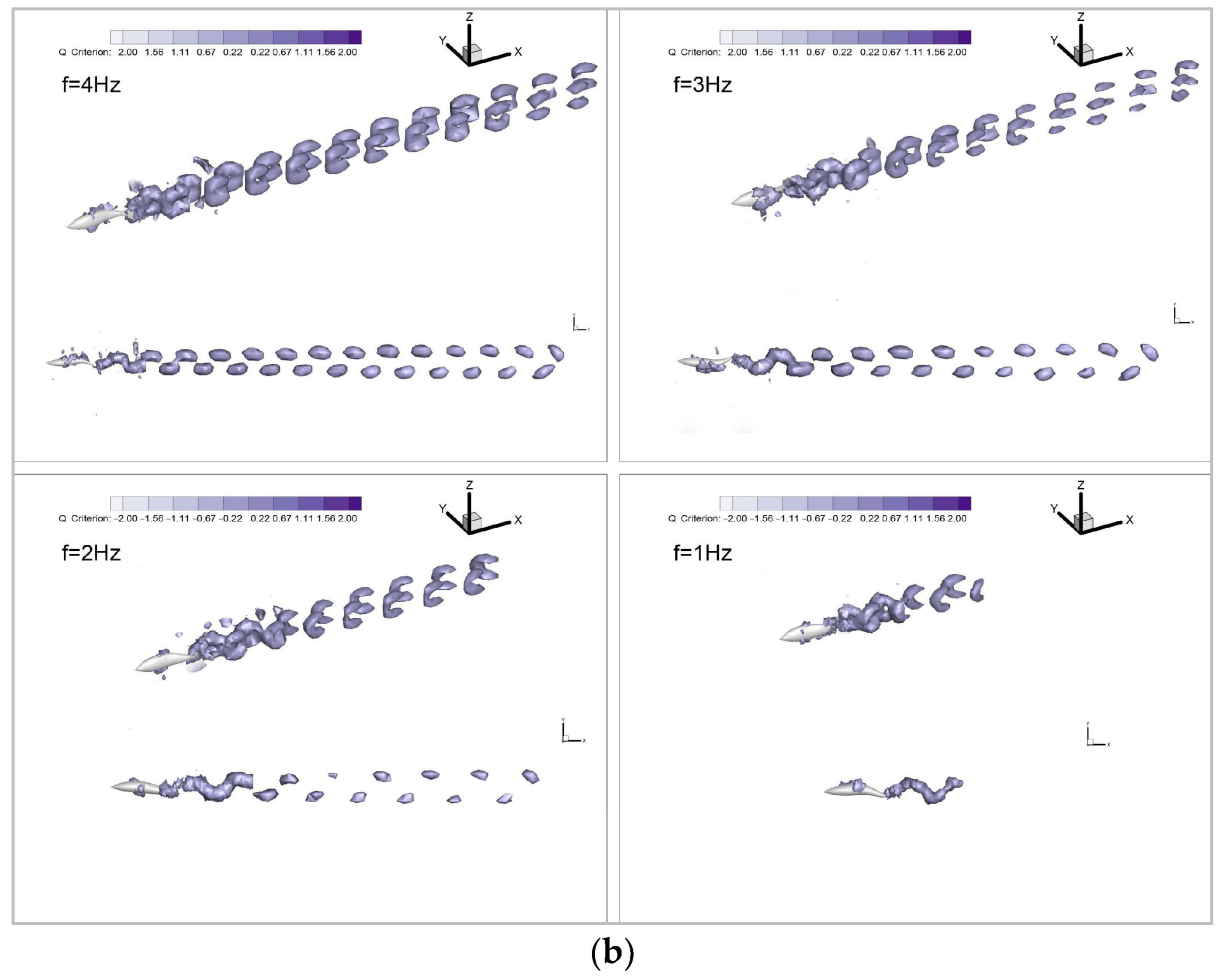
Vortex distribution of different wake-beat frequencies: (**a**) still water; (**b**) countercurrent waters.

To provide a more intuitive understanding of the influence of countercurrent perturbations on the efficiency and hydrodynamic characteristics of the biomimetic robotic fish Froude propulsion efficiency, we employ 3D Q-criterion isosurfaces (set at a value of 0.1) to visualize the flow dynamics around the swimmer, as depicted in Figure 9. Following the swimmer, wake vortices manifest in pairs, forming a characteristic reverse Kármán vortex street. Throughout the propulsive motion, these vortices gradually disperse toward the rear of the robotic fish on both sides, exhibiting a spread rather than clustering above or below the swimmer. Notably, the height of the vortex rings adjacent to the caudal fin closely matches the height of the fish itself. This unique flow-field configuration facilitates the induction of a jet effect by the reverse Kármán vortex street, generating a positive force applied to the swimmer and thereby enabling the biomimetic robotic fish to achieve high Froude efficiency. Consequently, the swimming mode of the biomimetic robotic fish demonstrates superior propulsive efficiency compared to the traditional propeller-driven mode.

## 4. Discussion

During long migrations, fish tend to seek out low-velocity zones as harbors for short rests and energy recovery [55]. Zhang et al. used particle image velocimetry, compared to acoustic Doppler velocimetry in shear flow, to characterize the turbulent velocity field downstream of four full-width prismatic obstacles [56]. The hydrodynamic relationship between the flow and the well-controlled swimming motion of a robotic fish was characterized by vorticity and tangential Reynolds stress fraction. In comparison to particle image velocimetry techniques, a CFD model was developed using a natural simulation approach to control the self-propulsion of the bionic robotic fish more accurately and closer to the swimming motion of real fish. The variation in the hydrodynamic coefficients of the bionic robotic fish in hydrostatic and countercurrent environments was intensively investigated and evaluated using Froude’s propulsive efficiency. Considering different tail-beating frequencies, a comparative analysis was conducted on the hydrodynamic coefficients and the Froude propulsion efficiency of the bionic robotic fish in still water and countercurrent environments.

Utilizing the established numerical simulation environment and rigorous post-processing of the simulation results, a comparative analysis is conducted to examine the hydrodynamic coefficient and Froude propulsion efficiency of the bionic robotic fish in hydrostatic and countercurrent water environments, considering varying tail-beat frequencies. Utilizing the established numerical simulation environment and rigorous post-processing of the simulation outcomes, we evaluate the Froude propulsion efficiency. Based on our calculations, it is definitively ascertained that the optimal efficiency is attained at tail-beat frequencies of *f* = 2.0 Hz and *f* = 3.0 Hz. Notably, it is observed that the bionic robotic fish is capable of augmenting its propulsive velocity by harnessing the force of water, particularly in certain countercurrent water environments.

Furthermore, a quantitative analysis is undertaken to scrutinize the anticameral vortex street of the bionic robotic fish, leveraging the post-processing software Tecplot. In contrast to the existing use of eddy currents to assess swimming performance, our results show that in static waters, tail-beat frequency is positively correlated with the lateral and longitudinal distances of the vortex nuclei within the antimatrix vortex street [57,58]. In countercurrent waters, the longitudinal distance *L_f_* is maximized for vortex nuclei at 3 Hz, whereas the transverse distance *a_f_* is minimized, thereby providing a visual validation of the variations in propulsion efficiency.

## 5. Conclusions

This paper aims to analyze the impact of various tail-beat frequencies on the hydrodynamic performance of biomimetic robotic fish. The study is conducted using numerical simulation methods in both hydrostatic and countercurrent waters. An autonomous motion model of the bionic robotic fish, powered by a function given by the user, is created. Additionally, a method for calculating the efficiency of its propulsion is shown. Tests and evaluations are conducted on the swimming performance of the biomimetic robotic fish in hydrostatic and countercurrent waters, considering various tail-beat frequencies. The findings indicate that the biomimetic robotic fish demonstrates the greatest effectiveness in propelling itself in waters flowing in the opposite direction, namely, at a tail-beat frequency of *f* = 3.0 Hz. Furthermore, the examination of isovorticograms of the flow field elucidates the process by which alterations in the aquatic environment impact the locomotion capabilities of the biomimetic robotic fish. These findings offer a crucial theoretical foundation for the development and enhancement of biomimetic robotic fish.

Despite some progress being made in the current research, there are numerous obstacles and prospects in investigating the hydrodynamic properties and propulsion effectiveness of biomimetic robotic fish. Through a thorough analysis of various factors and the utilization of advanced experimental techniques and numerical simulation methods, our ongoing work is to enhance the understanding of the locomotion mechanisms of biomimetic robotic fish. This will enable us to provide more efficient guidance and optimization for their use in challenging underwater environments. Follow-up research will persistently address these issues in the following areas to further advance the development and application of bionic robotic fish:

(i) The simplification of the robotic fish model was necessary to enhance computational speed. Although we considered the effects of tail-beat frequency and water-flow velocity on the locomotion of biomimetic robotic fish, we overlooked other factors that may influence hydrodynamic coefficients, such as the shape, size, and material properties of the robotic fish, as well as the role of pectoral fins in the propulsion process.
(ii) Despite the ability of numerical simulation to quantify various hydrodynamic values, its computational cost is high, and there are limitations to the scenarios that can be simulated.

To further the research, we propose the following measures:

(i) The comprehensive consideration of factors such as the shape, size, and material properties of biomimetic robotic fish, along with an in-depth analysis of the specific mechanisms of pectoral fins during propulsion. Additionally, advanced experimental techniques and numerical simulation methods can be employed to gain a more comprehensive understanding of the propulsion efficiency and hydrodynamic characteristics of biomimetic robotic fish.
(ii) The introduction of large-scale predictive modeling methods, where existing data can be utilized to establish datasets and combined with deep learning techniques for predictive analysis. This approach can significantly reduce computational costs while improving the accuracy and reliability of predictions.

## Figures and Tables

**Table 1:** Mesh sizes and simulation results.

Mesh Size	Skewness	Cells	Quality	Results (BL/s)	Error (%)
Coarse	0.75	204,596	0.799	0.62135	0.0296
Medium	0.91	238,512	0.789	0.60889	0.0091
Fine	0.88	260,727	0.837	0.60345	0

**Table 2:** Vortex street distribution in the flow field at different wake-beat frequencies in hydrostatic waters.

*f* (Hz)	*L_f_* (m)	*a_f_* (m)
4	0.9416	0.294
3	0.7998	0.294
2	0.4446	0.1970
1	0.4066	0.064

**Table 3:** Vortex street distribution in the flow field at different wake-beat frequencies in countercurrent waters.

*f* (Hz)	*L_f_* (m)	*a_f_* (m)
4	0.7	0.4926
3	0. 8	0.294
2	0.3	0.294
1	0.4	0.294

## Data Availability

Some or all of the data, models, or code that support the findings of this study are available from the corresponding author upon reasonable request.
